# Ginsenoside Rh7 Suppresses Proliferation, Migration and Invasion of NSCLC Cells Through Targeting ILF3-AS1 Mediated miR-212/SMAD1 Axis

**DOI:** 10.3389/fonc.2021.656132

**Published:** 2021-04-29

**Authors:** Xiangbo Chen, Wenguang Liu, Bao Liu

**Affiliations:** Key Laboratory of Molecular Epigenetics of the Ministry of Education (MOE), Northeast Normal University, Changchun, China

**Keywords:** ginsenoside Rh7, NSCLC, ILF3-AS1, miR-212, SMAD1

## Abstract

It is reported that ginsenosides have a significant anti-tumor effect on a variety of tumors. However, the role and mechanism of Rh7 in non-small cell lung cancer (NSCLC) are unclear. In this study, we aimed to study the anti-tumor effect of Rh7 on the proliferation and progression of NSCLC. Bioinformatics analysis showed that ILF3-AS1 was regulated by ginsenoside Rh7 in NSCLC. Down-regulation of ILF3-AS1 could significantly inhibit the proliferation, metastasis and invasion of NSCLC. In addition, ILF3-AS1 negatively controlled miR-212, which in turn targeted SMAD1 expression, thereby regulating NSCLC cell viability and apoptosis. Our results indicate that ILF3-AS1 can be used as a diagnostic and therapeutic target for non-small cell lung cancer. It is discovered for the first time that ginsenoside Rh7 inhibits the expression of ILF3-AS1 and exerts antitumor effects.

## Introduction

Non-small cell lung cancer (NSCLC) is one of the malignant tumors with the highest mortality rate worldwide (1). Platinum-based dual-drug combination chemotherapy or molecular targeted drugs for specific populations are the most common first-line treatment options for NSCLC patients, however, most patients with NSCLC will suffer the relapse within 1 year (2). Thus, identifying novel drugs for the treatment of NSCLC remains to be an urgent clinical need.

Ginsenosides have been reported to have significant anti-tumor effects on a variety of tumors including liver cancer, lung cancer, and gastric cancer (3). Ginsenoside can significantly inhibit tumor cell proliferation, invasion and metastasis, and reduce angiogenesis (4). For example, ginsenoside Rh2 monomer can inhibit the proliferation of cancer cells, induce tumor cell apoptosis, reverse the abnormal differentiation of tumor cells and suppress tumor metastasis (5, 6). Ginsenoside Rh1 affects the occurrence and development of liver cancer and malignant glioma and can inhibit tumor migration and invasion (7, 8). Ginsenoside monomer Rg5 can induce apoptosis in a variety of cancer cells, Rg5 extracted from black ginseng has been proven to promote apoptosis in human breast cells. A series of *in vitro* experiments confirm that ginsenoside Rg5 also has an inhibitory effect on the proliferation of esophageal cancer cells (9, 10). Exploring the functional roles of ginsenosides can provide novel clues for the identification of novel therapeutic methods for human cancers, including NSCLC.

Long non-coding RNA (lncRNA) is a class of RNA molecules longer than 200 nts, and it does not encode proteins (11). A large number of studies have shown that lncRNAs play an important regulatory role in human diseases, including cancers (12, 13). LncRNAs are found to be differentially expressed in a variety of tumors, including NSCLC (14). LncRNAs can specifically regulate the expression of downstream genes and participate in the regulation of tumorigenesis and progression, through regulating tumor cell proliferation, adhesion, migration, invasion, epithelial-mesenchymal transition and drug resistance (15). In NSCLC, a few studies have revealed the important regulatory role of lncRNAs in tumors (16–19). It has been found in NSCLC that high expression of lncRNA DLEU2 is associated with a shorter overall survival period, which can induce cell proliferation, invasion, migration and apoptosis (20). The expression of lncRNA GIAT4RA is significantly reduced in lung cancer tissues, and it inhibits the growth, colony formation, migration and invasion, EMT transformation, tumor globules and tumor growth of non-small cell lung cancer cells *in vivo (* 21). Related reports indicate that in many diseases, lncRNAs can be used as a competitive endogenous RNA (ceRNA) to regulate gene expression by sponging miRNAs. LINC81507 acts as a miR-199b-5p sponge to regulate the CAV1/STAT3 pathway, thereby inhibiting the occurrence and development of NSCLC (22). WGCNA is a powerful tool to understand the underlying mechanisms of human diseases. Long non-coding RNA (lncRNA) is a class of RNA molecules longer than 200 nts (23), which has been reported to play an important regulatory role in human tumors (24–26). Very interestingly, WGCNA has been widely used to understand the role of lncRNAs in human cancers. For example, Giuliet et al. used the WGCNA method to identify lncRNAs in pancreatic cancer and found that 11 lncRNAs were key regulators of pancreatic cancer (27).

In this study, we screened key novel ginsenosides that could extend a tumor-suppressive effect on NSCLC proliferation and progression, and we identified ginsenoside Rh7 could suppress NSCLC proliferation, migration and invasion. Bioinformatics analysis indicated ILF3-AS1 was regulated by ginsenoside Rh7 in NSCLC. Knockdown of ILF3-AS1 significantly affected NSCLC cell proliferation, migration, and invasion of NSCLC. Therefore, we provided a novel candidate drug and a putative new therapeutic target of it for NSCLC.

## Methods and Materials

### Materials and Reagents

Human NSCLC cell lines A549 and H1299 were obtained from American-type culture collection (ATCC, Manassas, VA, U.S.A.). CCK8 was obtained from Dojindo Laboratories (Kumamoto, Japan). Anti-SMAD1 (EP565Y) was purchased from Abcam.

### Cell Line Culture

Human NSCLC cell lines A549 and H1299 were cultured in DMEM with 10% FBS (BI, Israel) and 1% penicillin-streptomycin (BI, Israel). Keep the temperature at 37°C. The medium is updated 1-2 times a week.

### Cell Treatment

Ginsenosides Rc, Rh1, Rh3 and Rh7 were purchased from Beijing Solarbio Science & Technology Co., Ltd. (Beijing, China), dissolved in dimethyl sulfoxide (DMSO), and then diluted to a concentration range of 0-100μM. The cells were treated in ginsenoside for 24 hours.

Shanghai Gene Pharmaceutical Co., Ltd. (Shanghai, China) synthesized ILF3-AS1 and SMAD1 short interfering RNAs (siRNAs). A549 and H1299 cells were seeded into 6-well plates and transfected with siRNA or control siRNA (Genepharma Company, Shanghai, China) using Lipofectamine^®^ 2000 DNA transfection reagent (Invitrogen, Oregon, USA). The siRNA sequences were: si-ILF3-AS1-1, 5’-GTTCCTCTAAGTAATCGCCATGCGTTCT-3’, si-ILF3-AS1-2, 5’-UUUGUCCUUACAAGCGUGGTT-3’, si-SMAD1, 5’-CAGGACUUUGUGUACAGUUAA-3’, si-NC, 5’-UUCUCCGAACGUGUCACGUTT-3’.

### Transwell Migration and Invasion Assays

We used the transwell chamber (Corning, NY, USA) to conduct the assays of cell migration and invasion. The transwell chamber was coated with the matrigel mix (BD Biosciences, San Jose, CA, USA) for invasion assay, and it could also be conducted for migration assay with Matrigel mix coat.

A549 and H1299 cells at the logarithmic phase were digested and diluted to 1×10^5^/ml with the invasion medium of IMDM + 0.1% BSA without serum. About 100 µl cells were added on top of the transwell membrane in the upper chamber, and 600 µl of IMDM + 10% FBS + the related drugs were added to the lower chamber in a 24-well plate. After the drug treatment for 48 h, we used cotton swabs to scrape the cells settled on the upper surfaces of the transwell chambers and fixed the cells settled on the lower surfaces. The remaining cells were stained by DAPI. Next, we observed the number of cells in the transwell chambers under a fluorescent inverted microscope and took a photo later.

### Quantitative Real-Time PCR

TRIzol reagent (15596026, Invitrogen, Shanghai, China) was used to extract the total RNA samples from each tumor tissue, according to the manufacture’s introduction. 1 μg of total RNA was taken to reverse-transcribed cDNA using a MiRcute miRNA First-strand cDNA synthesis kit (Tiangen Biotech, Beijing, China) for miRNA, while the Primer-Script TM one-step real-time PCR reagent kit (Takara, Shiga, Japan) was used for the reverse transcription of lncRNAs and mRNAs, according to the manufacturer’s protocol. The relative quantitation of mRNA expression levels was measured using the SYBR Green I real-time PCR kit (CoWin Bioscience Co., Beijing, China). The sequences of all the primers were as follows: lncRNA ILF3-AS1: forward 5′-TAAACCCCACTGTCTTCC-3′, reverse 5′-TTCCTTGCTCTTCTTGCTC-3′; GAPDH (353bp), forward 5′-GGGAGCCAAAAGGGTCATCATCTC-3′; reverse 5′-CCATGCCAGTGAGCTTCCCGTTC-3′. In order to normalize the gene expression levels in each tumor tissue sample, the gene of GAPDH was used as an endogenous control for lncRNAs and mRNAs, whereas U6 was used as an internal reference for miRNAs. The changes in the mRNA expression in all the groups were calculated by the method of 2^−ΔΔCt^ (28).

### Cell Cycle Assays

The cells were harvested and washed with phosphate-buffered saline. The pellet was then resuspended, fixed in 70% pre-incubated methanol, and stored at 4°C overnight. The cells were washed again with PBS, and then a staining solution (propidium iodide) was added. Before flow cytometry analysis, the final mixture was incubated in the dark for 30 minutes. The experiment was performed three times and repeated three times. For cell cycle assay, G1, S and G2 peaks were detected from the propidium iodide-stained A549 and H1299 cells by flow cytometry (BD FACS Canto II, BD Bioscience, NJ, USA) and analyzed using the Modfit software.

### Dual-Luciferase Reporter Assay

The ILF3-AS1 (or SMAD1 3’UTR) was inserted into the full-length sequence psiCHECK2 basic configuration. We used Lipofectamine 3000 (Invitrogen, Cat# L3000-015), 0.5 µg reporter gene construct and 50 nm siRNA (or miRNA mimic) were transfected per well. After 12 hours of transfection, the transfection medium was replaced with a complete medium. After 48 hours of culture, the cells were lysed with passive lysis buffer (Promega, Cat# E1910), and the expression of the reporter gene was detected using the dual-luciferase reporter gene detection system (Promega, Cat# E1910). All transfection experiments were performed in 3 replicates.

### RNA Sequence

RNA-sequence (RNA-seq) was performed using BGI’s Illumina HiSeq 2500. We sent the Rh7-treated NSCLC cells to BGI Shanghai, got Generate-mat data in FASTQ, and aligned RNA-seq reads with human reference sequences. TopHat2 v2.0.14.27 STAR v2.5.2a28 for gene annotation (UCSC hg19) was used to calculate FPKM value. Bioinformatics analysis of ginsenoside Rh7 target in NSCLC applied DAVID system. The RNA-seq results were visualized using the Integrated Genome Viewer (IGV) tool of the Broad Institute.

### Statistical Analysis

The values presented in the study were represented as mean ± S.D. One-way ANOVA test followed by Dunett’s t-test was used as a calculated statistical method with SPSS19.0 statistical software. *, *P* < 0.05, **, *P* < 0.01 and ***, *P* < 0.001 were regarded as statistically significant.

## Results

### Screening of Ginsenosides That Played Tumor-Suppressive Roles in NSCLC

**Figure 1:**
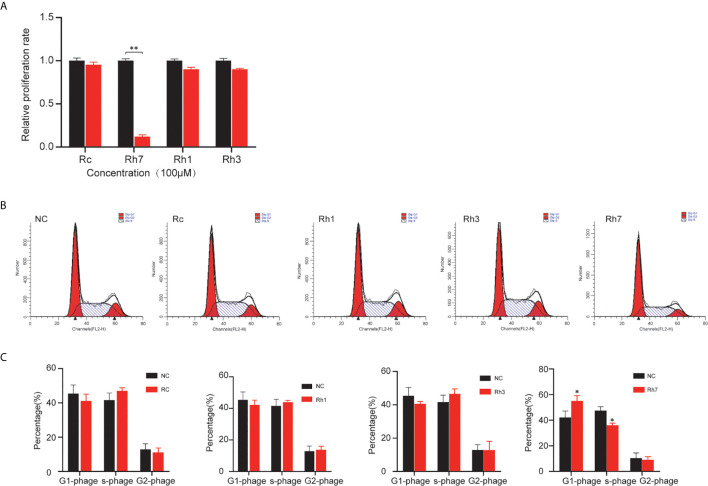
Effects of different ginsenosides on the proliferation ability of NSCLC cells. **(A)** The effect of different ginsenosides on the proliferation of H1299 cells determined by CCK-8 assay. **(B)** The effect of different ginsenosides on the cell cycle progress of H1299. **(C)** Statistical analysis of the decrease number in G0/G1 and S phase. *P < 0.05, **P < 0.01.

To identify ginsenosides’ regulatory role in NSCLC, we treated H1299 cells with 100 μM Rh7, Rh1, Rh3, and Rc. CCK-8 assay was performed to detect their effects on cell proliferation. As shown in **Figure 1A**, 100μM Rh7 could significantly inhibit H1299 proliferation. The H1299 cell growth treated with 100μM Rh7 was reduced by 83% compared with the control group.

Also, we investigated ginsenosides’ effect on the cell cycle in NSCLC cells. Cell cycle assay showed that Rh7 could significantly induce cell cycle arrest in H1299. However, Rh1, Rh3, and Rc had no significant effect on the H1299 cell cycle. H1299 in G0/G1 phase treated with 100 μM Rh7 increased by 30.8%, and H1299 in the S phase decreased by 29.8% compared with the control group (**Figures 1B, C**).

### Ginsenoside Rh7 Suppressed NSCLC Cell Proliferation, Migration and Invasion

**Figure 2:**
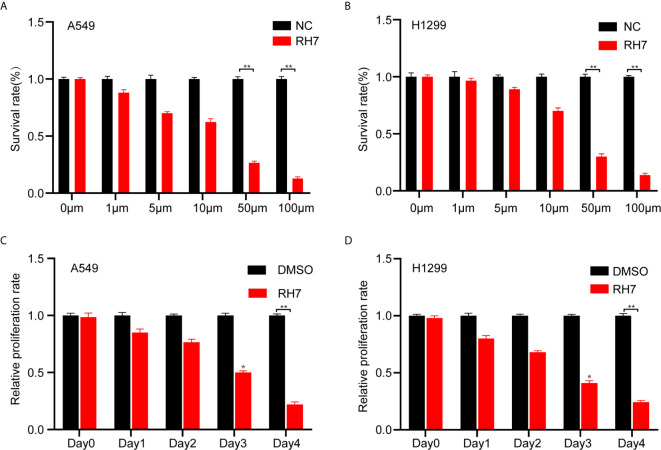
Effects of different concentrations of Rh7 on the survival rate of A549 and H1299 cells. **(A, B)** Effect of Rh7 on the survival rate of A549 and H1299 cells in the dose-dependent pattern. **(C, D)** Effect of Rh7 on the survival rate of A549 and H1299 cells in the time-dependent pattern.*P < 0.05, **P < 0.01.

To further validate the above findings and clarify the biological functions of ginsenoside Rh7, we used 0μM, 1μM, 5μM, 10μM, 50μM, 100μM Rh7 to treat A549 and H1299. The measured results were consistent with the results in the previous section. With increased Rh7 treatment concentration, the survival rate of A549 and H1299 showed an overall dose-dependent decrease. The half lethal dose of Rh7 for A549 was 22.5 μM (**Figure 2A**), and H1299 was 25.5μM (**Figure 2B**). Therefore, we chose to treat A549 and H1299 with 25μM Rh7.

Next, Rh7 effects on cell proliferation were examined in a time-dependent manner. A549 and H1299 were treated with 25 μM Rh7 for five days. As shown in **Figure 2**, Rh7 treatment significantly inhibited the growth rate of A549 and H1299. The growth rate of Rh7 treated A549 was reduced by 72% compared with the DMSO treatment group at day 4 (**Figure 2C**), and H1299 was reduced by 75% (**Figure 2D**).

**Figure 3:**
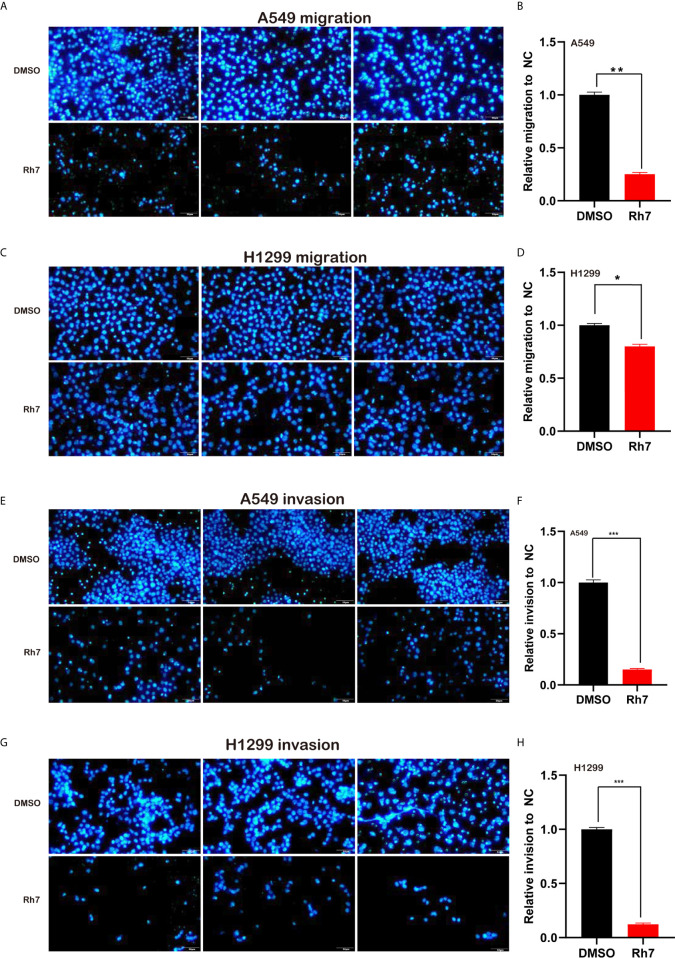
Rh7 treatment inhibits the migration and invasion ability of A549 and H1299 cells. **(A, B)** 25 μM Rh7 treatment inhibited the migration ability of the A549 cell line, and **(C, D)** 25 μM Rh7 treatment inhibited the migration ability of the H1299 cell line. **(E, F)** 25 μM Rh7 treatment inhibited the invasive ability of A549 cell line. **(G, H)** 25 μM Rh7 treatment inhibited the invasive ability of H1299 cell line. *P < 0.05, **P < 0.01, ***P < 0.001.

The effect of Rh7 on the migration ability of NSCLC cells was determined by transwell analysis. As shown in **Figure 3**, ginsenosides Rh7 could significantly inhibit NSCLC cell migratory ability. Compared with the control group, the migration number of Rh7-treated A549 decreased by 73.5% (**Figures 3A, B**), and H1299 decreased by 20.2% (**Figures 3C, D**). Very interestingly, we also found that Rh7 could significantly suppress the invasion of A549 (**Figures 3E, F**) and H1299 (**Figures 3G, H**).

### Ginsenoside Rh7 Induced a Repertoire of Differentially Expressed Genes

**Figure 4:**
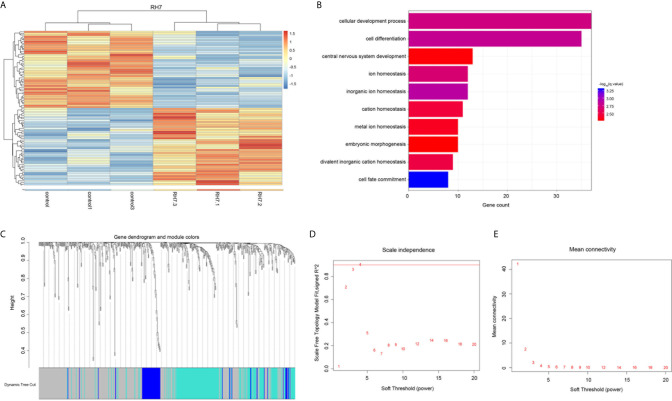
Bioinformatics analysis of the Rh7 regulated genes in NSCLC. **(A)** Heatmap diagram of the differentially expressed genes in H1299 cells is stimulated by ginsenoside Rh7. The abscissa represents genes and the ordinate represents sample names; red represents up-regulated expression and blue represents down-regulated expression; genes and samples are clustered separately. **(B)** GO analysis of differentially altered genes in H1299 cells stimulated by ginsenoside Rh7. **(C)** 496 clustering results. **(D)** The trend of scale independence when soft threshold is 1 to 20. **(E)** The trend of average connectivity when soft threshold is 1 to 20.

Functional assays showed that ginsenoside Rh7 could significantly inhibit the proliferation and cell cycle progression of NSCLC, however, the underlying mechanism remained to be unclear. Therefore, we detected the whole-genome gene expression profile after Rh7 treatment in NSCLC cells using RNA-seq technology. A total of 177 genes were found to be differentially expressed after Rh7 treatment in H1299 cells (**Figure 4A**). 87 genes were significantly up-regulated after Rh7 treatment, and 90 genes were down-regulated.

Next, we conducted the bioinformatics analysis of ginsenoside Rh7 targets in NSCLC using DAVID system. As shown in **Figure 4B**, Rh7-regulated genes were significantly involved in regulating multiple pathways, including cell development process, cell differentiation, central nervous system development, inorganic ion homeostasis, ion homeostasis, cation homeostasis, metal ion homeostasis, embryonic morphogenesis and bivalent inorganic cation homeostasis.

### WGCNA (Weighted Correlation Network Analysis) Analysis Uncovered Ginsenoside Rh7-Downstream Regulatory Genes

Gene weight co-expression analysis was conducted to further explore the mechanism of downstream regulatory genes of Rh7 in NSCLC. We constructed a gene-weighted co-expression network for Rh7 downstream regulatory genes using TCGA LUAD database. Hierarchical-clustering 576 tumor samples results were shown in **Supplementary Figure 1A**. No obvious abnormal values were found.

The fitting degree of the weighted co-expression network and the scale-free network obtained by using the WGCNA function pickSoftThreshold to detect an integer of 1-20 as a soft threshold was shown in **Figures 4D, E**. We chose the minimum soft threshold 4 whose scale-free network fit was higher than 0.9. Corresponding to **Supplementary Figure 1B**, we could see that the average degree of the weighted co-expression network at this time was also very low, which further proved that β=4 was suitable for the gene co-expression network built. When the minimum soft threshold was set to 4, the connectivity k of the constructed network also conformed to the Poisson distribution, that was the points with small connectivity account for the majority, while the points with large connectivity were rare, and there was no scale-free topology distribution (29). The combined degree R^^2^ also reached an optimal value of 0.89 (As shown in **Supplementary Figure 1C**).

After obtaining the soft threshold, we calculated TOM and performed hierarchical clustering to obtain the hierarchical clustering results of 496 Rh7 downstream regulatory genes (**Figure 4C**). A total of 3 gene modules were clustered, (blue module, turquoise module, grey module). Of the 496 genes, 262 genes were not classified into any one category, so they were regarded as a grey module separately. The remaining 234 genes were divided into 2 types of gene modules. Gene module 1 contained 178 genes and gene module 2 contained 56 genes.

Through the WGCNA clustering method, we obtained three gene modules (**Supplementary Figure 1D**). Although one of them was an obsolete module, its stability also affected the stability of the overall result, so the stability of the gene module needed to be further evaluated. First, clustering and correlation analysis were performed on the first principal component trend of each gene module, and the results were shown in **Supplementary Figure 1E**. From the clustering results, the clustering distance between the blue module and the turquoise module was very long, and the correlation of the first principal component was also low, only 0.12. This indicates that the trends of the two modules are almost uncorrelated. This also proves the stability of our WGCNA clustering results.

The overall module stability has been proven, then we should also evaluate whether the gene change trend in each module is consistent. As shown in **Supplementary Figure 1F**, the two-dimensional distribution of gene connectivity and gene significance in the blue and turquoise modules were also relatively stable, and the fitting trends within the two were different. The comparison between the correlation of the soft threshold conversion and the ordinary Pearson correlation without conversion also showed that although the correlation of the weight change after the soft threshold conversion was adjusted, the overall was more optimized. There were also different trends in the overall gene distribution, and the gene distribution in the turquoise module was wider. This shows that the clustering results are stable, and genes with different trends are clustered into different modules. Genes within the same module are more related and cluster more closely.

We also found an interesting phenomenon. Our results showed that non-coding RNAs might be widely involved in the regulation of downstream regulatory genes of Rh7. There were 4 lncRNAs (NCF1C, C7orf13, ILF3-AS1 (LOC147727), SNORA25, SNHG9) and 1 snoRNA (SNORA25) in the blue module; there were 2 lncRNAs (TP53TG1, RPPH1) and SNORA62 in the turquoise module. These lncRNAs will be the focus of our further investigation.

### Ginsenoside Rh7 Regulated the Expression of lncRNAs at the Core of the Network

**Figure 5:**
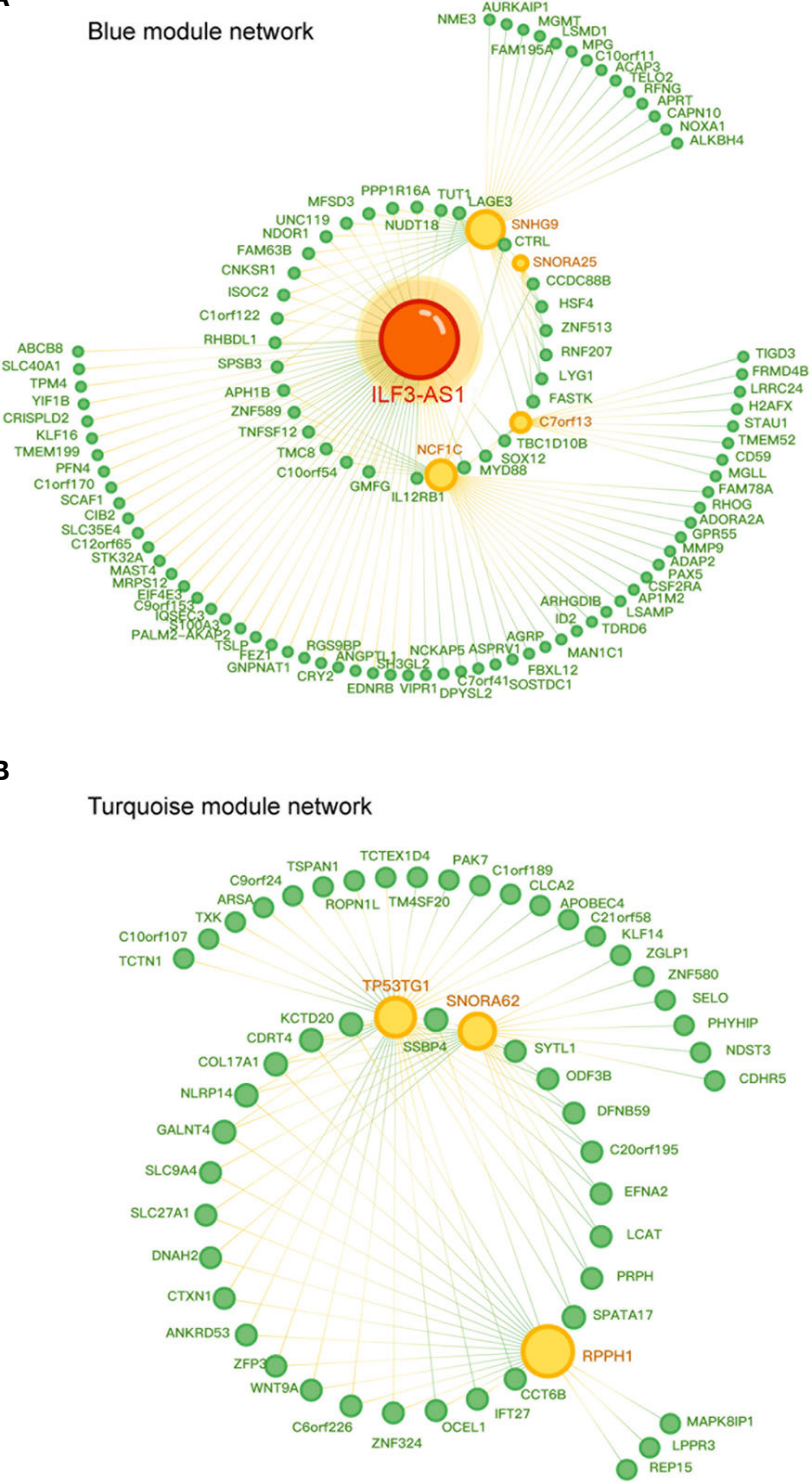
Co-expression network diagram of lncRNA-mRNA in the gene module. **(A)** Co-expression network diagram in the blue module, where green dots represent mRNA and yellow dots represent lncRNA. **(B)** Co-expression network diagram in Turquoise module, where green dots represent mRNA and yellow dots represent lncRNA. The size of a point depends on the connectivity of that point. The higher the connectivity, the larger the point.

Furthermore, we constructed a lncRNA-mRNA co-expression network in NSCLC by calculating the Pearson correlation coefficient of lncRNA-mRNA pairs in the two gene modules based on the WCGNA analysis. As presented in **Figure 5A**, the blue module-related network included 5 lncRNAs and 103 DEG, and the turquoise module-related network included 3 lncRNAs and 50 DEG (**Figure 5B**). Among these lncRNAs, we identified ILF3-AS1 as one of the key regulators in this network because it connected with 59 different nodes.

**Figure 6:**
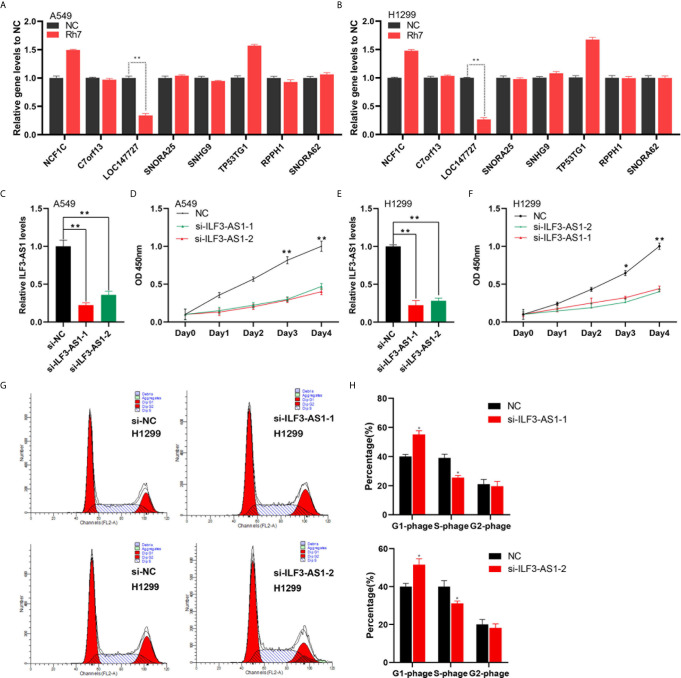
Knocking down of ILF3-AS1 reduced the growth rate of A549 and H1299 cells. **(A, B)** A549 and H1299 cell lines were treated with Rh7 to detect NCF1C, C7orf13, LOC147727, SNORA25, SNHG9, TP53TG1, RPPH1 and SNORA62 gene expression levels. **(C, F)** RT-PCR was used to detect ILF3-AS1 levels in non-small cell lung cancer cell lines A549 **(C)** and H1299 **(E)**. **(D, F)** Knockdown of ILF3-AS1 significantly suppressed cell proliferation in non-small cell lung cancer cell lines A549 **(D)** and H1299 **(F)**. **(G, H)** Knockdown of ILF3-AS1 could significantly block the cell cycle progression of H1299. *P < 0.05, **P < 0.01.

In order to validate the effect of Rh7 on the key lncRNAs expression in the network, qRT-PCR assay was conducted for A549 and H1299 cells after Rh7 treatment. It was worth noting that the expression of ILF3-AS1 showed the most significant change after treatment with Rh7. ILF3-AS1 expression decreased by 66% in A549 cells (**Figure 6A**), and by 73.3% in H1299 cells (**Figure 6B**), after treated with Rh7.

### Knockdown of ILF3-AS1 Significantly Inhibited NSCLC Cell Proliferation, Migration and Invasion

To investigate the functional roles of ILF3-AS1 in NSCLC, we designed two siRNAs (si-ILF3-AS1-1 and si-ILF3-AS1-2) to target ILF3-AS1. After transfection, ILF3-AS1 level was significantly reduced in both A549 (**Figure 6C**) and H1299 cells (**Figure 6E**).

Next, we investigated the biological functions of ILF3-AS1 in NSCLC. We found knockdown ILF3-AS1 significantly inhibited the growth rate of A549 (**Figure 6D**) and H1299 cells (**Figure 6F**). The A549 cell growth was reduced by 53% and 60% after transfection with siRNA-1 and siRNA-2 respectively, and H1299 was reduced by 56% and 58.2%.

In this study, we also detected ILF3-AS1 effect on the cell cycle of A549 and H1299. The results showed that knockdown ILF3-AS1 significantly blocked the cell cycle of H1299. The cells in the G0/G1 phase of the ILF3-AS1 knockdown group significantly increased in number, while the proportion of cells in the S phase and G2/M phase decreased compared with the control group in the lung cancer cell line (**Figures 6G, H**).

**Figure 7:**
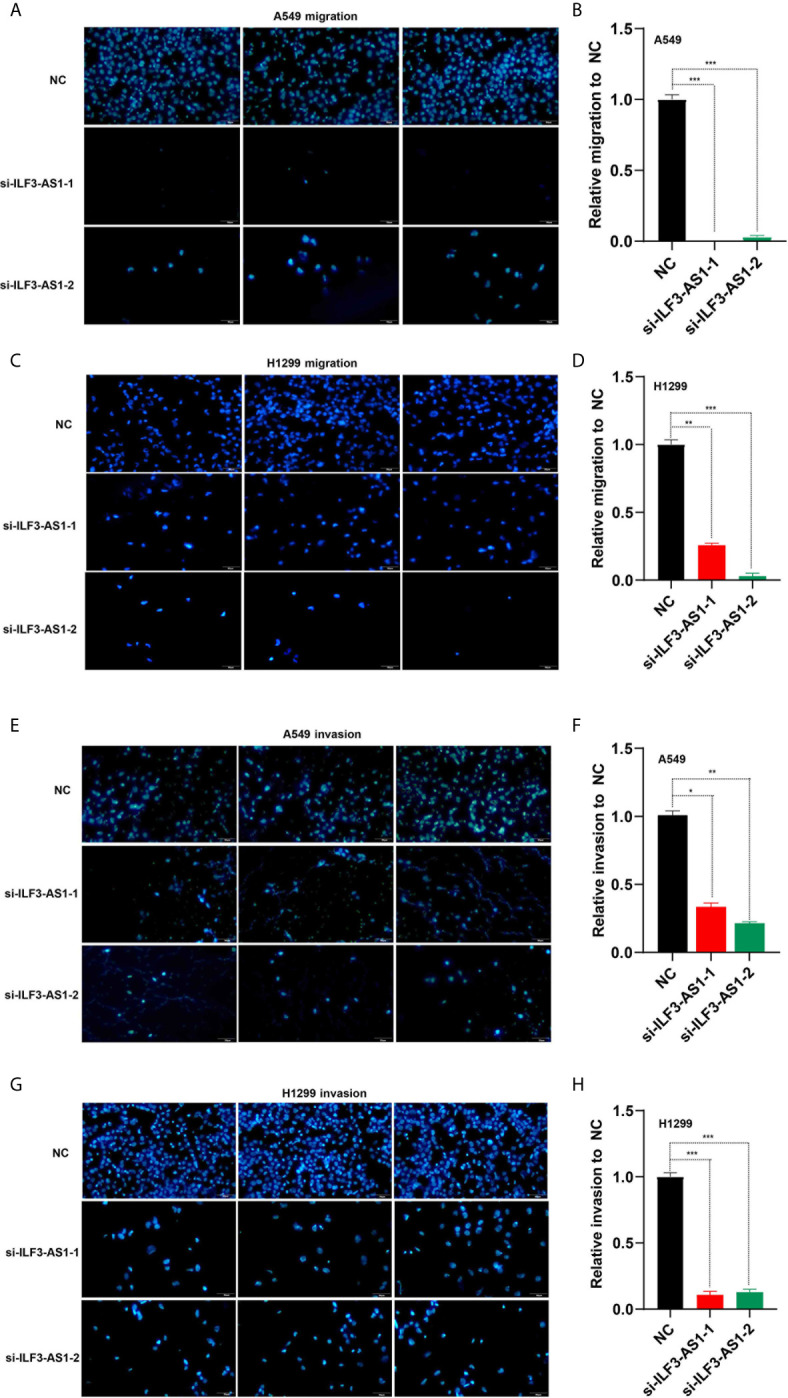
Knockdown of ILF3-AS1 inhibited the migration and invasion capacity of A549 and H1299 cell lines. **(A, B)** Knockdown of ILF3-AS1 inhibited the migration ability of A549 cell lines. **(C, D)** Knockdown of ILF3-AS1 inhibited the migration ability of H1299 cell lines. **(E, F)** Knockdown of ILF3-AS1 inhibited the invasion ability of A549 cell lines. **(G, H)** Knockdown of ILF3-AS1 inhibited the invasion ability of H1299 cell lines. *P < 0.05, **P < 0.01, ***P < 0.001.

The effect of ILF3-AS1 on the migration and invasion capacity of NSCLC cells was next determined. After knocking down ILF3-AS1 in A549 and H1299, compared with the control group, the cell migration (**Figures 7A–D**) and invasion (**Figures 7E–H**) was significantly reduced.

### Construction of ILF3-AS1 Mediated Competing Endogenous RNA (ceRNA) Networks in NSCLC

The results of the co-expression network analysis showed that ILF3-AS1 could significantly regulate 59 potential mRNAs. Next, we used the DIANA-lncBase (https://bigd.big.ac.cn/databasecommons/database/id/336) and TARGETSCAN database (http://www.targetscan.org/vert_72/) to predict ILF3-AS1 targeting miRNAs and revealed 5 miRNAs that potentially binded to ILF3-AS1, including hsa-miR-320d, hsa-miR-7-5p, hsa-miR-212-3p, hsa-miR-4725-5p and hsa-miR-504-5p. We constructed a ceRNA network that mediated the regulation of ILF3-AS1 on downstream targets in NSCLC using the Starbase database. As shown in **Supplementary Figure 2**, a total of 5 miRNAs, 59 mRNAs and ILF3-AS1 were included in this network.

**Figure 8:**
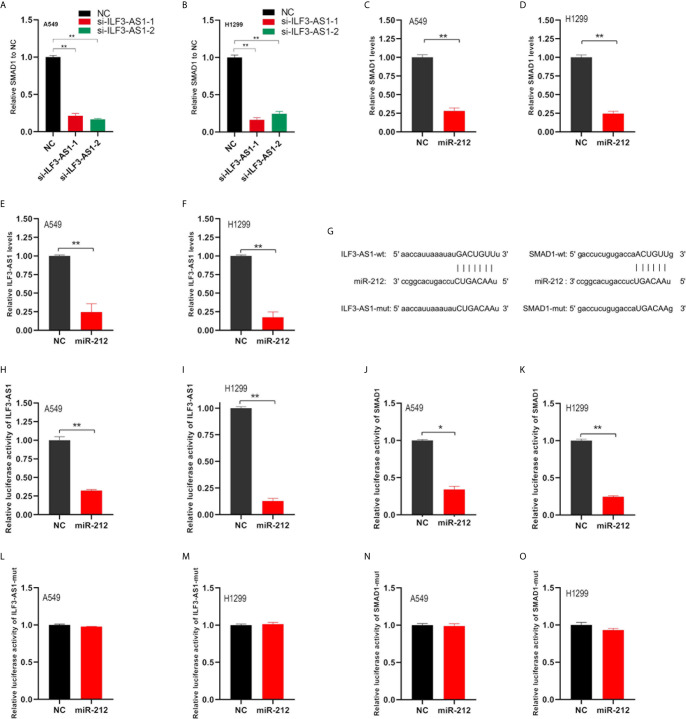
ILF3-AS1 and SMAD1 are both target genes of miR-212. **(A, B)** RT-PCR was used to detect SMAD1 levels in non-small cell lung cancer cell lines A549 **(A)** and H1299 **(B)** after knockdown of ILF3-AS1. **(C, D)** RT-PCR was used to detect SMAD1 levels in non-small cell lung cancer cell lines A549 **(C)** and H1299 **(D)** after overexpression of miR-212. **(E, F)** RT-PCR was used to detect ILF3-AS1 levels in non-small cell lung cancer cell lines A549 **(C)** and H1299 **(D)** after overexpression of miR-212. **(G)** Schematic diagram of the binding sequence of miR-212 and ILF3-AS1, miR-212 and SMAD 1-3’UTR predicted by online website. **(H, I)** miR-212 significantly inhibited intracellular luciferase activity of ILF3-AS1 (compared to miR-NC). **(J, K)** miR-212 significantly inhibited intracellular luciferase activity of SMAD1 (compared to miR-NC). **(L, M)** miR-212 did not inhibit intracellular luciferase activity of ILF3-AS1-mut (compared to miR-NC). **(N, O)** miR-212 did not inhibit intracellular luciferase activity of SMAD1-mut (compared to miR-NC). *P < 0.05, **P < 0.01.

In order to verify the ILF3-AS1 effect on its potential ceRNA targets, we used TargetscanHuman to predict the potential target genes of miR212 and prioritize the predicted targets. Besides, we found that knockdown ILF3-AS1 resulted in a significant decrease in SMAD1 gene level in NSCLC cells (**Figures 8A, B**). Therefore, SMAD1 was selected as the main target of miR212 for further study. SMAD1 protein was involved in the regulation of the TGF-β signaling pathway, which was reported to be abnormally expressed and involved in regulating cancer metastasis of multiple cancers, including NSCLC.

### ILF3-AS1 and SMAD1 Were Both Target Genes of miR-212

Next, we verified whether SMAD1 and ILF3-AS1 were targets of miR-212. First, we found miR-212 overexpresion could significantly inhibit the levels of SMAD1 (**Figures 8C, D**) and ILF3-AS1 (**Figures 8E, F**). Subsequently, we used the TargetScan and miRDB databases to find the complementary pairing sequence of miR-212 to SMAD1-3’UTR and ILF3-AS1 (**Figure 8G**). Dual-luciferase reporter assay was further applied to validate the interaction between miR-212 and SMAD1-3’UTR or ILF3-AS1. We found that miR-212 remarkably inhibited the luciferase activity of ILF3-AS1-wt (**Figures 8H, I**) and SAMD1-wt (**Figures 8J, K**) plasmid, but not ILF3-AS1-mut (**Figures 8L, M**) and SAMD1-mut (**Figures 8N, O**) plasmid.

### Highly Expressed SMAD1 and ILF3-AS1 Were Significantly Positively Related to the Shorter Survival Time of Patients With NSCLC

**Figure 9:**
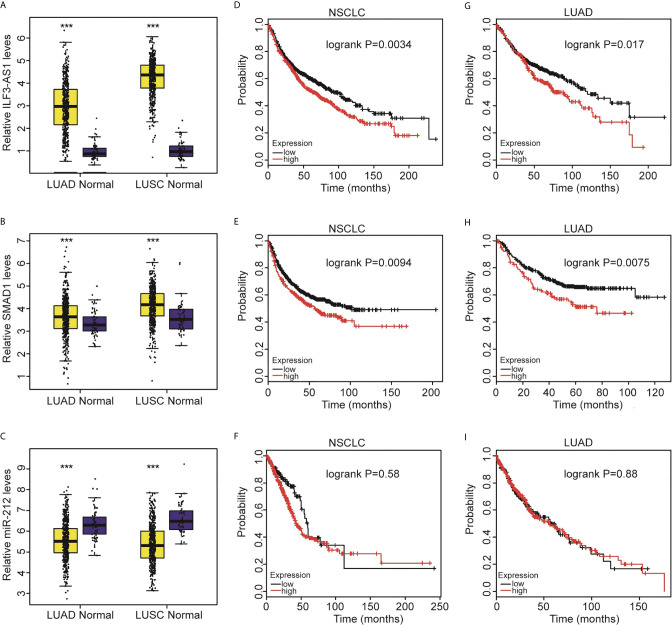
Highly expressed SMAD1 and ILF3-AS1 were significantly positively related to the shorter survival time of patients with non-small cell lung cancer. Compared with normal tissues, SMAD1 **(A)** and ILF3-AS1 **(B)** were significantly higher expressed in LUAD and LUSC, while miR-212 **(C)** was significantly lower expressed in LUAD and LUSC. In NSCLC, the higher expression of SMAD1 **(D)** and ILF3-AS1 **(E)** showed a significant positive correlation with shorter patient survival time, while miR-212 expression had no significant correlation with patient survival time. **(F)**. The high expression of SMAD1 **(G)** and ILF3-AS1 **(H)** in LUAD showed a significant positive correlation with shorter patient survival time, while miR-212 **(I)** expression had no significant correlation with patient survival time. ***P < 0.001.

To investigate the prognostic value of SMAD1, ILF3-AS1, and miR-212 for NSCLC patients, we analyzed TCGA dataset. ILF3-AS1 levels (**Figure 9A**) and SMAD1 (**Figure 9B**) in NSCLC samples were significantly higher than those in normal samples, but miR-212 (**Figure 9C**) was significantly lower in NSCLC than in normal tissues.

Next, we used the TCGA database to analyze the association of SMAD1, ILF3-AS1 and miR-212 expressions with the overall survival of NSCLC. We found that higher expression of ILF3-AS1 (**Figures 9D, G**) or SMAD1 (**Figures 9E, H**) was significantly correlated with shorter overall survival time for both NSCLC and LUAD patients (**Figures 9F, I**). However, we did not find a significant prognostic value of miR-212 expression for NSCLC and LUAD patients.

**Figure 10:**
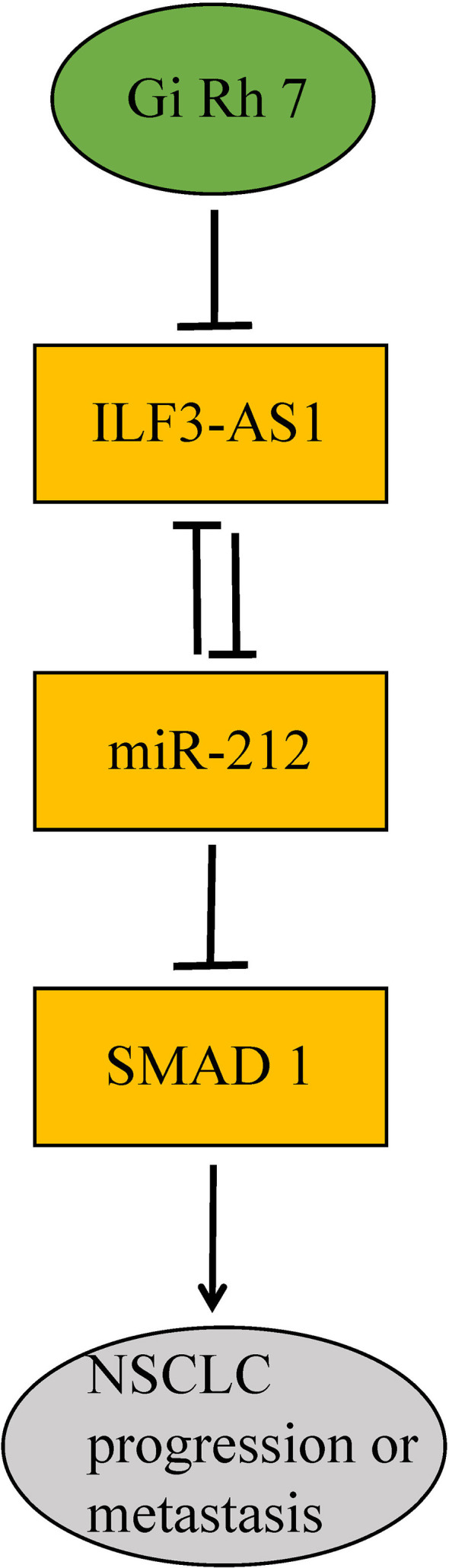
Diagram of the mechanism of ginsenoside Rh7 targeting the ILF3-AS1/miR212/SAMD1 axis in NSCLC.

In conclusion, this study for the first time showed Ginsenoside Rh7 can suppress the expression of ILF3-AS1, which could competitively bind with miR-212, thus leading to the down-regulation of SMAD1 and suppression of NSCLC progression (**Figure 10**).

## Discussion

Ginsenosides have been reported to have important anti-tumor roles in a variety of tumors including liver cancer, lung cancer, and gastric cancer (30). Ginsenoside Rhs is revealed to inhibit the metastasis and proliferation of cancer cells (31, 32). For example, Rh1 could significantly inhibit the metastatic ability of liver cancer and gliomas (33). In gliomas, Rh1 can inhibit the invasion and migration of U87MG cells by inhibiting the activity of ERK, JNK and p38 pathways (34). Rh3 is reported to down-regulate the expression of cell cycle regulators, cyclin E and Cdk2, thereby blocking the G1 phase of the cell cycle to suppress cell proliferation (35, 36). Of note, the molecular functions of Rh7 in cancer cells remain unclear. In this study, we found that Rh7 could significantly inhibit the proliferative and metastatic potentials of NSCLC. Among the 5 different Ginsenosides, we found that 100 μM Rh7 could significantly inhibit the proliferation of cells and block the progress of the cell cycle. Further study revealed that Rh7 could also significantly suppress cell migration and invasion. Thus, our results for the first time showed that Rh7 might serve as a potential anti-tumor and anti-metastasis drug for NSCLC.

In this study, we further explored Rh7 downstream regulatory genes in NSCLC. We constructed a gene-weighted co-expression network for Rh7 downstream regulatory genes based on TCGA LUAD dataset and identified two gene modules that played an important regulatory role in lung cancer. Interestingly, our results showed that lncRNAs, such as NCF1C, C7orf13, ILF3-AS1 (LOC147727) and SNORA62, played a crucial role in the regulation of downstream regulatory genes of Rh7.

In many cases, lncRNAs are the main regulators of gene expression and can play a key role in various biological functions and disease processes, including cancer. The lncRNA ILF3-AS1 is overexpressed in a variety of human cancers (37, 38). For example, it is revealed that ILF3-AS1 together with 14 lncRNAs predicts the survival of patients with cervical cancer (39). Bioinformatics analysis reveals that ILF3-AS1 is involved in the regulation of colon cancer proliferation, angiogenesis and cell death (40). In melanoma, lncRNA ILF3-AS1 is up-regulated in tumor tissues and is associated with poor patient prognosis (41). Our study for the first time shows that knockdown of ILF3-AS1 can significantly inhibit the proliferation, migration and invasion of NSCLC cells.

Recently, several reports have shown that ginsenosides can regulate lncRNAs to play an important role in anti-cancer activity. For example, in breast cancer cells, Ginsenoside Rh2 can inhibit the expression of C3orf67-AS1 through promoter methylation and exert an anti-proliferative effect (42). Ginsenoside Rg3 inhibits the migration and invasion of colorectal cancer cells by inhibiting the expression of lncRNA CCAT1, and promotes their apoptosis (43). In this study, we initially explored the regulatory mechanism of Rh7 on lung cancer cells. It was discovered for the first time that ginsenoside Rh7 could regulate cell proliferation, migration and invasion by inhibiting ILF3-AS1 expression in NSCLC cells. ILF3-AS1 should be the pharmatheutical target of ginsenoside Rh7. We found that Rh7 treatment could significantly suppress the expression of ILF3-AS1 in NSCLC cells.

MiRNA is a type of ncRNA with a length of about 22 nts. Mature miRNAs can regulate target mRNAs by binding to miRNA response elements (MREs) on the 3′-untranslated region (3-UTR) of the target mRNAs (44). CeRNA hypothesis points out that mRNA, pseudogene, circular RNA (circRNA), lncRNAs can act as ceRNA to regulate each other’s expression by completely binding to MREs (45). The present study shows that ILF3-AS1 is localized in the cytoplasm of NSCLC, suggesting that it may function as an endogenous competitive RNA. Interestingly, studies in osteosarcoma have shown that ILF3-AS1 can competitively bind miR-212 to promote the SOX5 axis, and thus enhancing the proliferation, migration and invasion of osteosarcoma cells (37). In this study, we constructed a ceRNA network and found ILF3-AS1 could affect SMAD1 expression through miR-212 in NSCLC. Knockdown of ILF3-AS1 or overexpression of miR-212 could significantly inhibit SMAD1 gene expression. And we found that miR-212 could significantly inhibit the luciferase activity of the wild-type SMAD1-3’UTR-reporter plasmid and the ILF3-AS1-reporter plasmid, but did not affect that of the mutant ones. These results demonstrated that SMAD1 and ILF3-AS1 were targets for miR-212.

Related reports indicate that miR-212 is a tumor suppressor, which is found in cervical cancer, pituitary tumors, bladder cancer and NSCLC. In NSCLC, Incoronato et al. found that miR-212 negatively regulated the expression of the apoptotic protein PED/PEA-15 (46). Smad protein is considered to be a major regulator of TGF-β and BMP signaling pathways, which can regulate cell growth and differentiation (47). In colorectal cancer, Smad1 promotes tumor cell migration by inducing Snail expression but has no significant effect on Twist1 expression (48). Therefore, there may be a miR-212-Smad1 axis in cancer. Ginsenoside Rh7 can regulate the expression of ILF3-AS1, ILF3-AS1 competitively binds with miR-212 in turn, and the miR-212-Smad1 axis could be enhanced by ginsenoside Rh7, thus ginsenoside Rh7 could significantly inhibit the proliferation, migration and invasion of NSCLC cells (**Figure 10**).

This study has limitations. It is necessary to further explore the expression levels of ILF3-AS1, miR-212 and SMAD1 in clinical samples. In future studies, we will collect more clinical samples to explore the correlation between the expression of ILF3-AS1, miR-212 and SMAD1 and clinical parameters (including gender, age, and clinical stage). In subsequent studies, we will use a variety of assay methods, such as RNA immunoprecipitation and RNA pull-down assays, to further verify the direct or indirect interaction of ILF3-AS1, miR-212 and SMAD1 in NSCLC.

In summary, our results for the first time show that ginsenoside Rh7 can significantly inhibit the proliferation and metastatic potentials of NSCLC cells ILF3-AS1/miR-212/SMAD1 axis. Bioinformatics studies have shown that Rh7 is widely involved in the regulation of ion homeostasis, monocarboxylic acid transport, neuroactive ligand-receptor interaction pathways, and cAMP signaling pathways. Furthermore, a co-expression network analysis shows that the long non-coding RNA ILF3-AS1 is widely involved in the regulation of ginsenoside Rh7 downstream networks. These results show that ILF3-AS1 can be used as a diagnostic and therapeutic target for NSCLC. Ginsenoside Rh7 inhibits the expression of ILF3-AS1 and exerts anti-tumor effects.

## Data Availability Statement

The datasets presented in this study can be found in online repositories. The names of the repository/repositories and accession number(s) can be found below: https://www.biosino.org/OES070088.

## Author Contributions

Conception and design: XC, WL and BL. Manuscript and revision: XC, WL and BL. All authors contributed to the article and approved the submitted version.

## Conflict of Interest

The authors declare that the research was conducted in the absence of any commercial or financial relationships that could be construed as a potential conflict of interest.

## Supplementary Material

Supplementary Figure 1 WGCNA analysis of ginsenoside Rh7-downstream regulatory genes **(A) **576 clustering results. **(B)** Histogram distribution of simulated network connectivity. **(C)** The fitness trend simulation of the network. **(D)** Cluster analysis between gene modules. **(E)** Principal component analysis and correlation analysis between gene modules. **(F)** The comparison between weight correlation and Pearson correlation after soft threshold processing. The abscissa represents the weight correlation and the ordinate represents the Pearson correlation.

Supplementary Figure 2 This PPI contained 5 miRNAs, 59 mRNAs and ILF3-AS1.
