# Supplementary figures and images for: Ginsenoside Rh7 Suppresses Proliferation, Migration and Invasion of NSCLC Cells Through Targeting ILF3-AS1 Mediated miR-212/SMAD1 Axis
Source: Front Oncol. 2021 Apr 29;11:656132. doi: 10.3389/fonc.2021.656132 (PMC8116958; doi:10.3389/fonc.2021.656132)

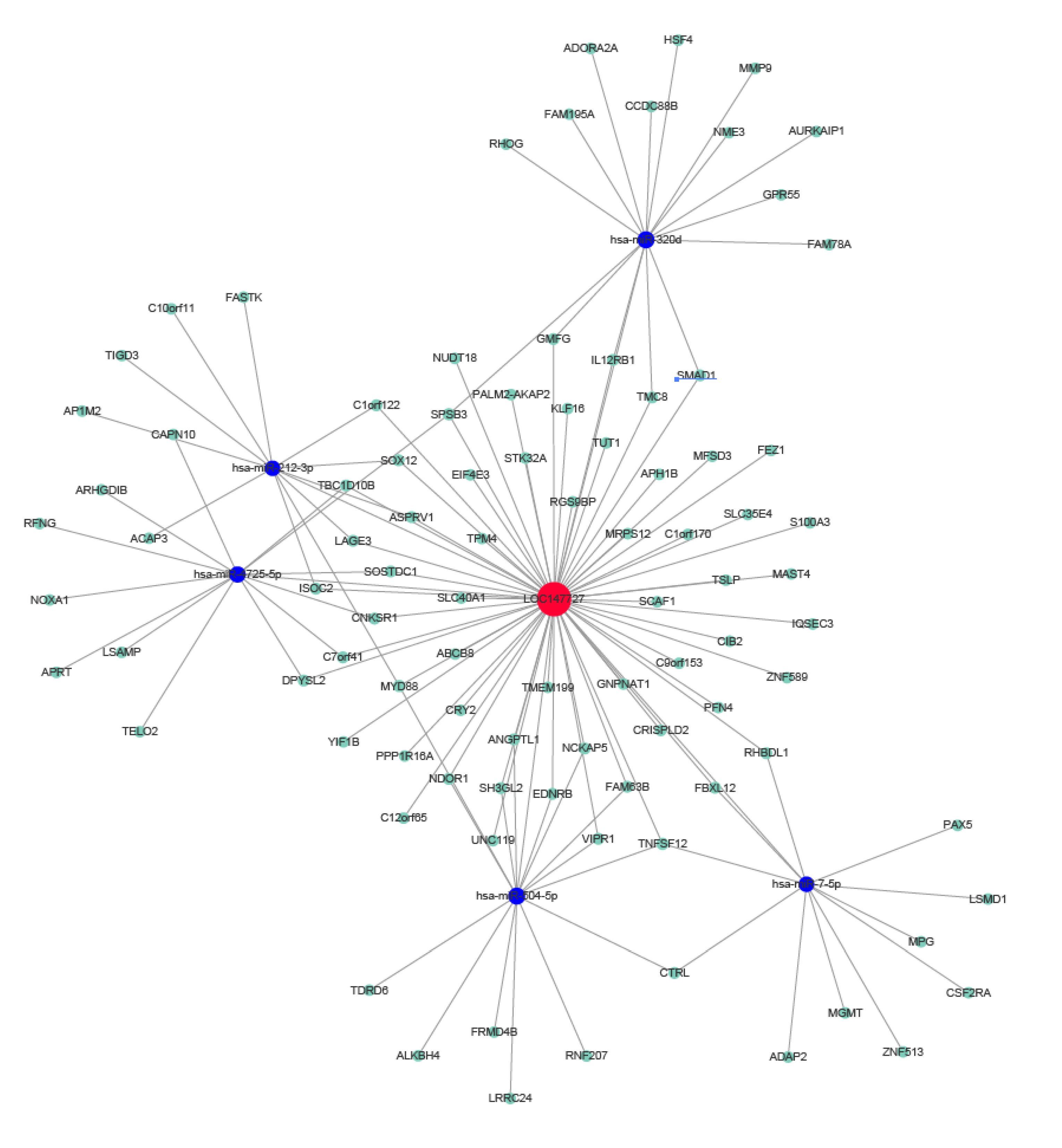

Supplement: Supplementary Figure 2 — This PPI contained 5 miRNAs, 59 mRNAs and ILF3-AS1. [file Image_2.tif]
